# Relative biological effectiveness and neural stem cell fate in carbon ion–irradiated human brain organoids

**DOI:** 10.1016/j.radonc.2025.111224

**Published:** 2025-10-24

**Authors:** Tamara Bender, Marco Durante, Insa S. Schroeder

**Affiliations:** a GSI Helmholtzzentrum für Schwerionenforschung, Biophysics Department, Darmstadt, Germany; b Institute for Condensed Matter Physics, Technische Universität Darmstadt, Darmstadt, Germany; c Department of Physics „Ettore Pancini“, University Federico II, Naples, Italy

**Keywords:** Particle therapy, Carbon ions, Brain lesions, Cerebral organoids, Choroid plexus

## Abstract

**Background and purpose:**

Heavy ion therapy offers a promising approach for treating brain tumors due to the high precision of ion beams and biological effectiveness. However, the mechanisms underlying contrast-enhanced brain lesions, which occur more frequently after ions than conventional X-rays, remain to be elucidated.

**Materials and methods:**

Cerebral organoids were generated from human embryonic stem cells and exposed to high-energy carbon-ions at early (day 20) and late (day 80) developmental stages. Analyses of gene expression were performed using RT-PCR analysis, and protein expression was assessed using immunofluorescence staining.

**Results:**

We demonstrate that exposure to high-energy carbon (C)-ions induces the formation of ectopic choroid plexus (CP)-like structures in human cerebral organoids. RT-PCR analyses revealed that C-ion exposure alters the differentiation trajectories of neuroepithelial stem cells, driven by dysregulation of key developmental signaling pathways including NOTCH, WNT, and BMP. Moreover, we observed increased expression of CP–associated markers AQP1 and CLDN3. The relative biological effectiveness (RBE) of C-ions for the formation of fluid-filled cavities associated with CP-like tissue was calculated as 2.0 after both 60 and 100 days of differentiation.

**Conclusion:**

Our findings indicate that aberrant CP development is a central mechanism underlying post-radiation contrast-enhanced brain lesions, and that an RBE of 2 should be considered when determining dose thresholds for normal tissue tolerance in carbon ion therapy for brain tumors.

## Introduction

Brain tumors represent a significant clinical challenge due to their aggressive nature and the need to preserve critical functions of the surrounding healthy brain tissue. Effective treatment often requires a combination of surgery, chemotherapy, and radiation therapy. Yet, conventional X-ray radiotherapy can inadvertently damage normal brain tissue, leading to complications such as cognitive deficits and radiation-induced brain lesions (as reviewed in [1]). Due to the more efficient irradiation utilizing carbon ions and protons relative to conventional X-ray therapy, the number of brain tumor patients undergoing these advanced treatment modalities has steadily increased in recent years [2,3]. Particle therapy has gained increasing attention in treating brain tumors due to its ability to deliver highly conformal dose distributions, thereby minimizing damage to surrounding healthy tissue. Among the modalities, carbon (C) ion therapy offers distinct physical and biological advantages, including a reduced lateral scattering and enhanced relative biological effectiveness (RBE) compared to protons [4,5]. Despite these benefits and technical improvements, particle therapy can still induce adverse effects in normal brain tissue, underscoring the need for a more comprehensive understanding of its safety profile. In particular, the pathophysiological mechanisms underlying post-irradiation contrast-enhanced lesions (CELs), generally attributed to radiation necrosis (RN), remain poorly characterized, even though such lesions are a frequent complication following particle therapy [6–9]. Although protons have a great potential to spare normal brain tissue [10], pediatric brain tumor patients treated with proton therapy have exhibited unexpectedly high rates of RN [11,12], as well as a range of other radiation-induced lesions [6]. Emerging studies suggest that ionizing radiation can disrupt neurodevelopmental processes [13], yet the relationship between early molecular events and radiologically visible lesions remains insufficiently characterized, creating a critical barrier to understanding and mitigating post-therapy complications. A major challenge in addressing this issue is the lack of appropriate models that effectively mimic the complexities of normal brain tissue. Human organoids provide a valuable model to investigate radiation-induced effects in a physiologically relevant setting [14–17]. These 3D structures can recapitulate key features of the brain, including neural progenitor cells, mature neurons, and the regional identity and organization of different brain regions [18]. This makes brain organoids an ideal tool for mechanistic studies on how radiation affects normal brain tissue. Although their use can also involve ethical and safety considerations [19], they can potentially reduce the reliance on animal models and complement animal studies. However, the absence of vasculature, diverse cell types, and the complex architecture of the human brain, hinders the modeling of interactions of neural cells with vessels and other brain or systemic components. Nevertheless, it allows us to focus on specific cell types and mechanisms relevant to the onset of CELs. In our previous study, we reported that both X-ray and proton irradiation result in the formation of aberrant CP-like structures in human brain organoids, as a form of radiation-induced tissue remodeling [20]. This was more pronounced in younger organoids than in more mature organoids, being in accordance with existing clinical data demonstrating the high susceptibility of pediatric brain tumor patients to radiation-induced brain lesions after proton therapy [6]. However, epidemiological data on the risk of such lesions after carbon ion radiotherapy for pediatric brain cancers is scarce (reviewed in [21]). In the present study, we focus on the effects of high-energy carbon ions, both for the molecular mechanism and to estimate the relative biological effectiveness (RBE) for brain toxicity. We hypothesize that carbon ions lead either to decrease in the formation of aberrant CP-like structures or to the similar effects observed after X-rays and protons.

## Materials and methods

### Cell culture & organoid generation

Cerebral organoids were generated from H9 cells following established unguided differentiation protocol according to Lancaster et al. [18], allowing a spontaneous differentiation of neural progenitors (See Supplementary Materials and Methods). All experiments were conducted using the human embryonic stem cell line WA09-FI (H9) under ethical approval by Central Ethics Committee for Stem Cell Research according to §4 and §6 of the German Stem Cell Act (registry numbers 3.04.02/0125 and 3.04.02/0125-E01).

### Irradiation procedure

Cerebral organoids were subjected to carbon ion irradiation in a dose range of 0.5–4 Gy or 3–15 Gy. Irradiation was performed at two distinct developmental stages—day 20 (d20), when the organoids primarily consist of neuroepithelial progenitor cells (immature stage), and day 80 (d80), when a mature neuronal network has formed—to assess stage-specific responses to radiation.

### Timepoints and analysis

Organoid size and cavity formation in cerebral organoids were analyzed for radiation-induced abnormalities. Images were captured using a standard digital camera (Sony DSC-W220), and organoid areas were quantified using ImageJ software. Additionally, the number of organoids displaying visible cavities was recorded for organoids at day 60 (irradiated at day 20) and organoids at day 100 (irradiated at day 80), respectively, and expressed as a percentage of the total number analyzed. Putative changes in gene and protein expression during the cultivation period up to 100 days (or 160 days in some cases) were determined using qRT-PCR and immunofluorescence (IF) analysis.

### Molecular assays

#### IF analysis

For IF analysis, organoids were fixed in 3.7 % formaldehyde (Carl Roth), dehydrated in a sucrose gradient (7–60 % sucrose in PBS; Sigma), embedded in 7.5 % gelatin (Neolab)/10 % sucrose, snap-frozen on dry ice and stored to −80 °C prior to cryosectioning. Following permeabilization of cryosections with 0.5 % Triton X-100/1% BSA and blocking with 1 % BSA, they were incubated with diluted primary antibodies, washed in PBS, and then incubated with diluted secondary antibodies. Imaging was performed on a Zeiss Axio Imager Z2 fluorescence microscope equipped with Metafer5 software (v4.3.12, Metasystems). Image processing was carried out using ImageJ (v1.53i, NIH).

#### Quantitative RT-PCR

For the analysis of the gene expression at the mRNA level, organoids were lysed in QIAzol Lysis Reagent (Qiagen, #79306). Total RNA was extracted using the RNeasy Mini Kit Qiagen, #74106), following the manufacturer’s protocol. Isolated total RNA was reverse transcribed using the RevertAid RT Kit (Life Technologies, #K1691). Quantitative PCR was performed using the Hot FIREPol EvaGreen qPCR Mix Plus (Solis Biodyne, #08–24–0000S) on a QuantStudio 3 Real-Time PCR System (Applied Biosystems), with data analysis carried out using QuantStudio Design & Analysis software (v1.5.3). Expression levels were normalized to 18S rRNA, and human fetal and adult brain mRNA samples were included as reference controls.

#### Statistics

Experiments were performed using organoid samples in triplicate (n = 3) for at least two independent experiments (N = 2). Where applicable (N = 3), statistical significance was determined using a threshold of p < 0.05. Statistical analyses were performed using appropriate ANOVA tests for number of independent experiments (N) with a given number of organoids per experiment (n) as indicated in the figure legend.

A comprehensive description of all experimental procedures, including cell culture conditions, organoid generation, irradiation parameters, immunostaining protocols, RNA extraction, qPCR details, and statistical methods, is available in the Supplementary Material.

## Results

**Fig. 1.**
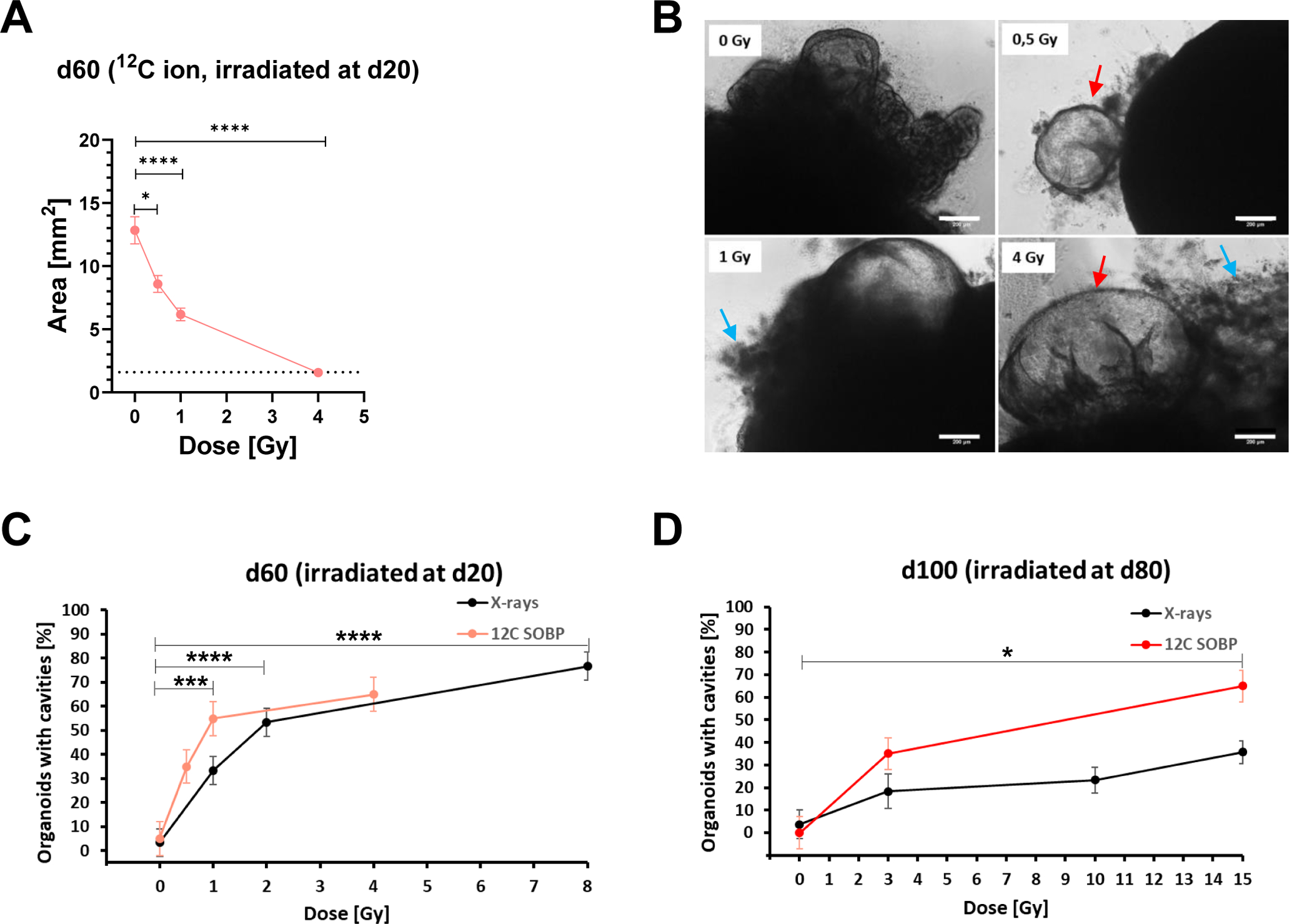
Growth retardation and formation of fluid-filled cavities in brain organoids after ^12^C irradiation. A: Size of cerebral organoids at d60 of the culture irradiated at d20 with 0, 0.5, 1, and 4 Gy measured as area in mm^2^ (N = 3, n = 10). The dotted line represents the value for d20 control organoids. Statistical analysis was done by Brown-Forsythe and Welch ANOVA with Dunnetťs post-test. Data is presented as mean ± SEM for three independent experiments (N = 3) and 10 organoids per experiment. B: Representative images of d60 organoids with morphological changes after irradiation with 0, 0.5, 1 and 4 Gy at d20. Cell decay (blue arrows), cavities (red arrows). Scale bar: 200 μm. C-D: Dose-dependent cavity formation as percentage of organoids displaying cavities, analyzed at d60 (C) and d100 (D) of the culture, respectively, for organoids irradiated at d20 and d80, respectively (N = 3, n = 10 per experiment (^12^C-ion, d20); N = 3, n = 25–36 (X-rays, d20); N = 1–2, n = 10 (^12^C-ion, d80); N = 4, n = 40–54 (X-rays, d80). Statistical analysis was done by Brown-Forsythe and Welch ANOVA with Dunnetťs post-test where applicable (N ≥ 3). The error bars represent the standard deviation (SD). D: in culture flasks at d60 following irradiation at day 20 with 0, 0.5, 1, and 4 Gy. Data is presented as mean ± SD for three independent experiments (N = 3) or as a single experiment (N = 1) and n = 10 organoids per variant/group. Data for X-rays were previously published [20]. (For interpretation of the references to colour in this figure legend, the reader is referred to the web version of this article.)

Irradiation of cerebral organoids with C-ions in the SOBP at d20 of the culture caused a progressive, dose-dependent growth retardation by d60 of the culture (Fig. 1A). The non-irradiated organoids at d60 showed an increase (8.5-fold) in size (circular area) compared to d20 controls, while the organoids exposed to 0.5 Gy exhibited a 1.5-fold decrease in size compared to the respective non-irradiated control at d60 (Fig. 1A). The organoids irradiated with 1 Gy showed a 2.2-fold decrease in size, and those irradiated with 4 Gy 8.2-fold decrease compared to the non-irradiated control at d60, with a size reduced to the size of control organoids at d20 (Fig. 1A).

Liquid-filled cavities developed in organoids exposed to different doses of C-ions as exemplary shown for d60 organoids irradiated at d20 of the culture (Fig. 1B). The increase in the percentage of organoids with liquid-filled cavities was dose-dependent, and it was observed at both d60 (40 days post-irradiation at d20) and d100 (20 days post-irradiation at d80) (Fig. 1C-D). While only 5 % of the organoids in the non-irradiated controls at d60 formed cavities, up to 65 % of the organoids developed cavities at d60 at the highest dose (Fig. 1C). No cavities were observed in control organoids at d100 (Fig. 1D). Irradiation with 3 Gy C-ions led to cavity formation in ~ 35 % organoids, and 15 Gy in ~ 60 % organoids (Fig. 1D). In organoids irradiated at d20 (Fig. 1C) or d80 (Fig. 1D), C-ions in the SOBP were more effective in inducing cavity formation than X-rays at the same dose, with an RBE of 2. For organoids irradiated with C-ions at d20 (Fig. 1C), the cavity formation in ~ 50 % organoids was a linear response only at doses up to 1 Gy. The slope of the fitted dose–response curves and the RBE of C-ion beams are reported in Table 1 and compared to the proton RBE measured in our previous work [20].

**Fig. 2.**
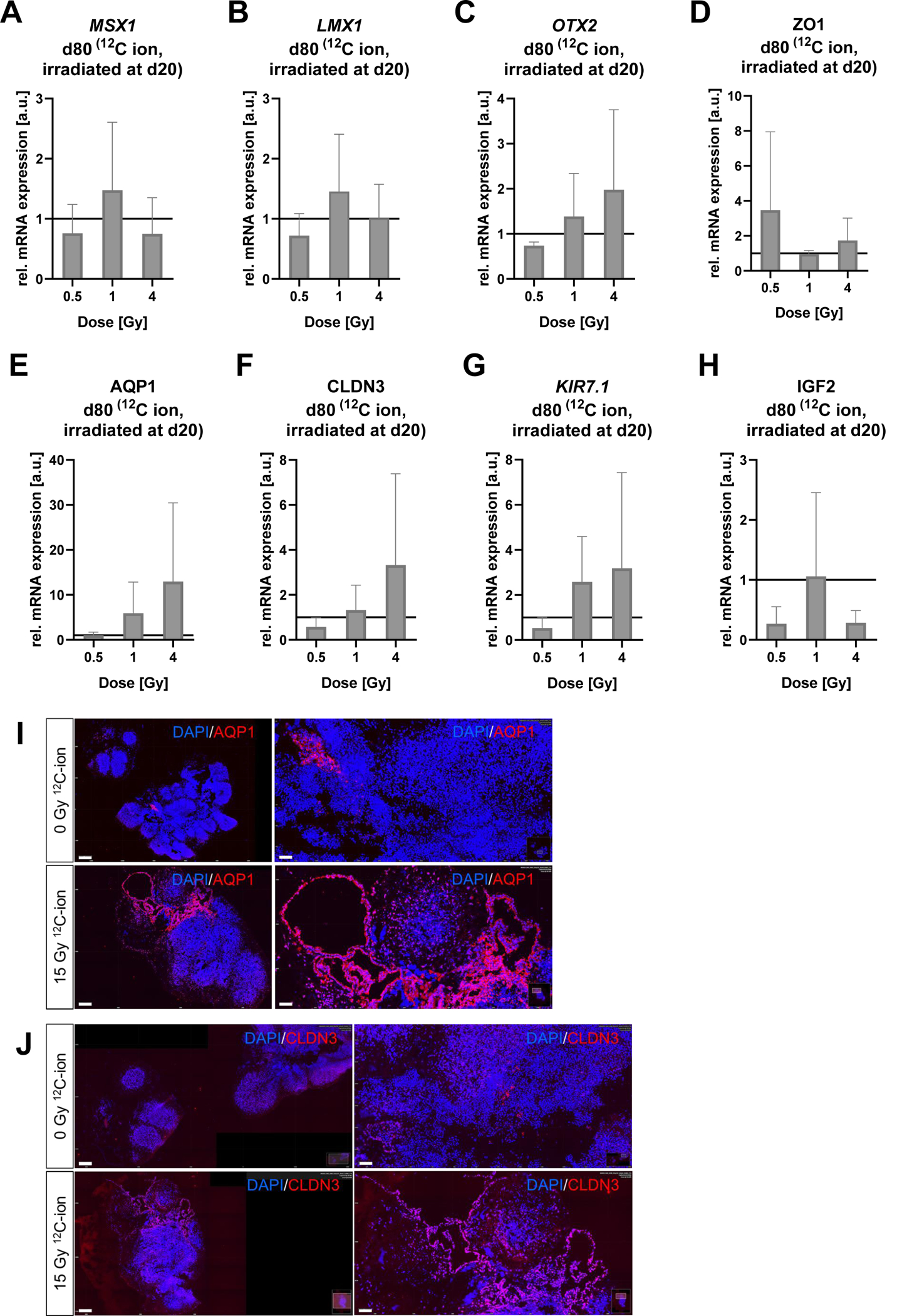
Formation of the choroid plexus in brain organoids after ^12^C irradiation. A-H: Relative mRNA expression of the CH and CP lineage marker *MSX1* and *LMX1A*, choroid epithelium marker *OTX2*, CP markers *ZO1*, *AQP1*, *CLDN3*, *IGF2* and *KIR7.1* in d80 organoids subjected to 0.5–4 Gy ^12^C-ion irradiation at d20, mean ± SD for at least two independent experiments (*N* = 2), otherwise N = 1, and *n* = 3 organoids per experiment. Values were normalized to 18S rRNA levels and expressed relative to values for the sham-irradiated control. The lines indicate the levels in the sham-irradiated controls. Statistical analysis was done by Welch ANOVA with Dunnetťs post-test where applicable (N = 3). The error bars represent the standard deviation (SD). I-J: Representative immunofluorescence staining of the CP markers AQP1 (red) and CLDN3 (red), and nuclei stained with DAPI (blue) in d100 organoids, scale bar = 100 μm or 200 μm for overview images, and 20 μm or 50 μm for magnification images. (For interpretation of the references to colour in this figure legend, the reader is referred to the web version of this article.)

A modest dose-dependent upregulation in the expression of cortical hem (CH) and choroid plexus markers after irradiation was revealed by real-time qPCR analyses (Fig. 2A-C). *MSX1*, which instructs the differentiation of the CP [22] and CP lineage marker *LMX1A* [22] were elevated at the mRNA expression level after 1 Gy, but not after 0.5 Gy and 4 Gy, respectively, compared to the sham-irradiated controls (Fig. 2A-B). Choroid epithelium marker *OTX2* [23] was elevated after 1 Gy and 4 Gy, with no change after 0.5 Gy (Fig. 2C). The mRNA level of the tight junction protein marker (*ZO1*) found in the CP epithelium [24] was only increased after 0.5 and 4 Gy compared to the sham-irradiated controls (Fig. 2D). The CP markers including water channel aquaporin 1 (*AQP1)*, tight junction protein claudin 3 (*CLDN3*, both specific for the CP epithelium [24]), and inwardly rectifying potassium channel *(KIR7.1),* expressed mainly in secretory cells of the CP [25,26], were elevated at the mRNA expression levels after 1 and 4 Gy, but not after 0.5 Gy, compared to non-irradiated organoids at day 80 (Fig. 2E-G). Insulin-like growth factor (IGF) 2, highly enriched in CSF and expressed during brain development [27], was increased after 1 Gy compared to the control at day 80 (Fig. 2H). However, these increases were not statistically significant (p > 0.05) due to the large variance of the data.

Nevertheless, a specific and persistent CP-like structure was present as a monolayer of epithelial cells in irradiated organoids. Immunofluorescence confirmed robust induction of CP-like epithelial structures in organoids exposed to 15 Gy C-ion irradiation at d80 and analyzed at d100, with strong AQP1 and CLDN3 expression lining fluid-filled cavities, a feature rarely observed in sham controls (Fig. 2I-J).

**Fig. 3.**
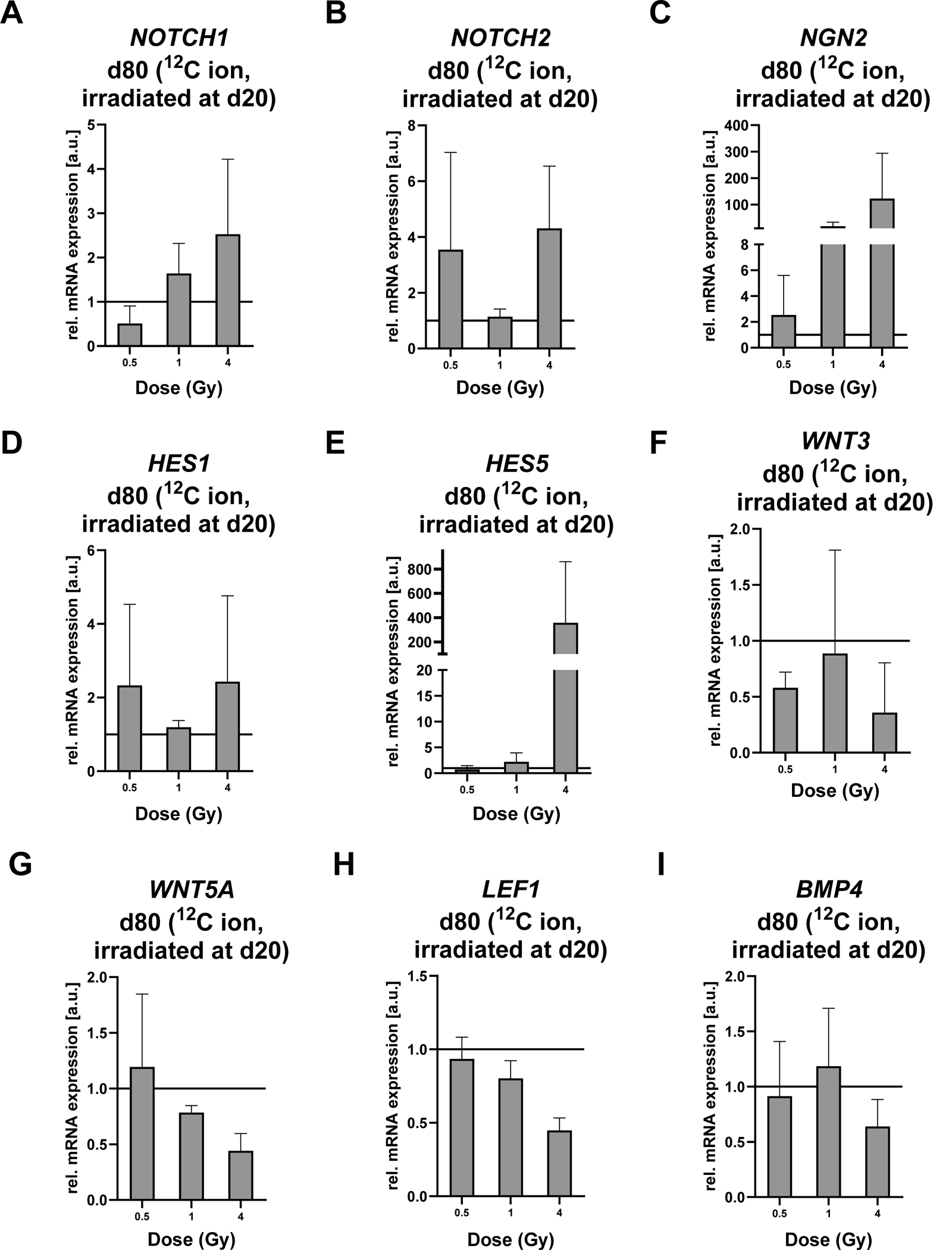
NOTCH/HES and WNT/BMP signaling in cerebral organoids after ^12^C-ion irradiation. A-I: Relative mRNA expression of the markers NOTCH1, NOTCH2, NGN2, HES1, HES5, WNT3, WNT5a, LEF1 and BMP4 in d80 organoids subjected to 0.5–4 Gy ^12^C ion irradiation at d20, mean ± SD for at least two independent experiments (*N* = 2), otherwise N = 1, and *n* = 3 organoids per experiment. Values were normalized to 18S rRNA levels and expressed relative to values for the sham-irradiated control. The lines indicate the levels in the sham-irradiated controls. Statistical analysis was done by Welch ANOVA with Dunnetťs post-test where applicable (N = 3). The error bars represent the standard deviation (SD).

A modest, not statistically significant (p > 0.5), dose-dependent change in neurogenesis-related signaling pathways was observed after C-ion irradiation as an increase in the mRNA expression of members of the NOTCH, WNT, and BMP signaling pathways that regulate neurogenesis and the CP formation [28–30]. *NOTCH1* and *HES5* mRNA levels were upregulated in d80 organoids irradiated at day 20 with 1 and 4 Gy C-ion compared to the non-irradiated controls, while 0.5 Gy resulted in a decrease or no change (Fig. 3A, E). *NOTCH2* and *HES1* mRNA levels were upregulated following irradiation with a dose of 0.5 and 4 Gy, respectively, or showed almost no change after 1 Gy (Fig. 3B, D). *NGN2* mRNA levels were upregulated regardless of the dose of irradiation (Fig. 3 C). *WNT3*, *WNT5A*, and *LEF1* mRNA levels remained mostly unchanged or decreased after irradiation (Fig. 3F-H), except for *WNT5A*, which was increased after 0.5 Gy (Fig. 3G). *BMP4*, which is regulated by *WNT3* [23], was only upregulated upon irradiation with 1 Gy, while there was a decrease in the mRNA level after 1 Gy and 4 Gy C-ion irradiation (Fig. 3I).

**Fig. 4.**
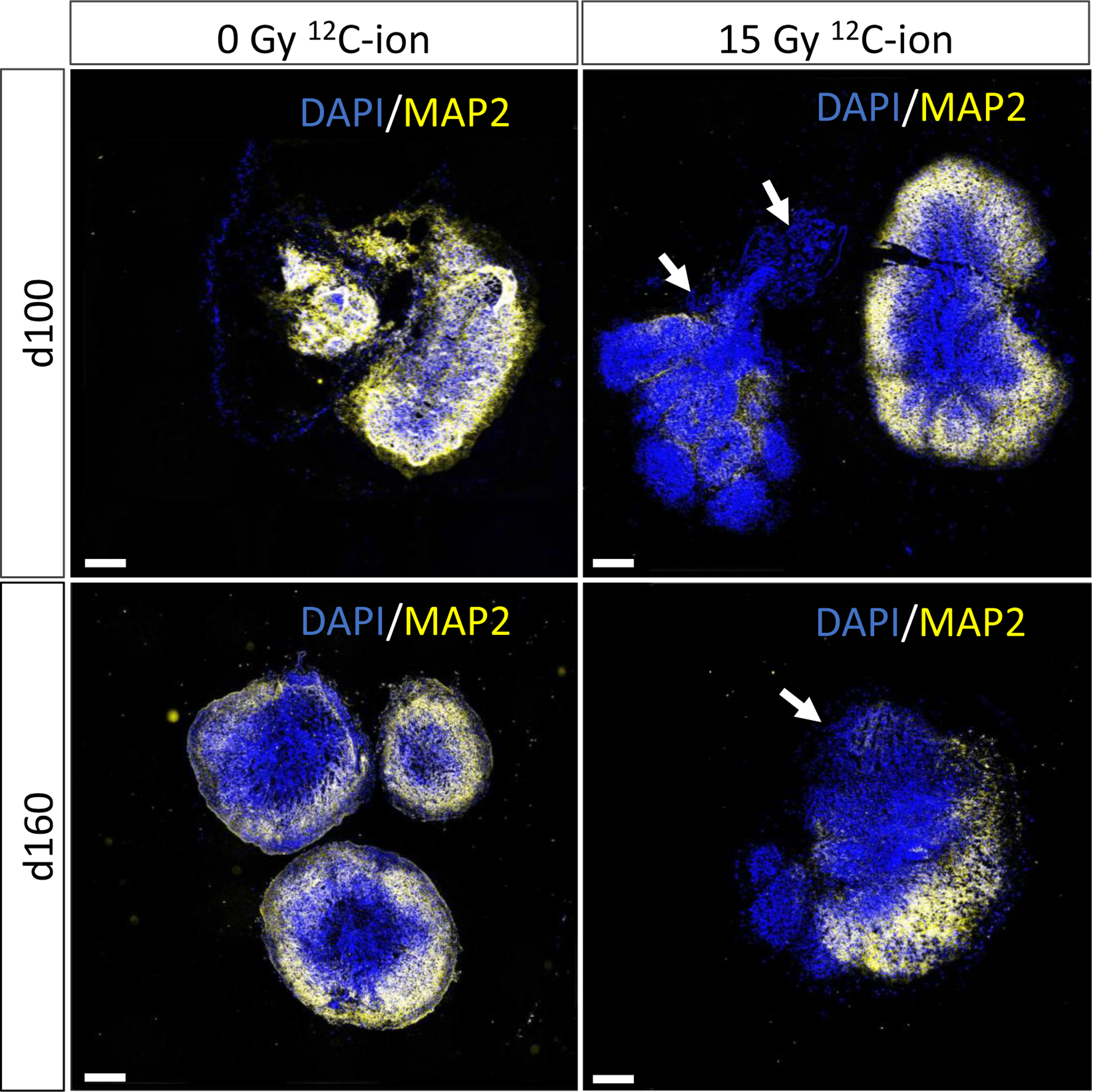
Neuronal cells in brain organoids after ^12^C irradiation. Representative immunofluorescence staining of the microtubule-associated protein 2 (MAP2) (yellow), and nuclei stained with DAPI (blue) in d100 and d160 organoids, respectively; choroid plexus-like structure indicated with arrows, scale bar = 100 μm. (For interpretation of the references to colour in this figure legend, the reader is referred to the web version of this article.)

A decrease in the expression of neuronal marker microtubule-associated protein 2 (MAP2) was observed in organoids exposed to 15 Gy C-ions irradiated at d80, and analyzed at d100 and d160, respectively (Fig. 4). This decrease in the MAP2 expression was restricted to areas of irradiated organoids displaying CP-like structure, which was rarely observed in sham-irradiated controls.

## Discussion

We have proposed the use of cerebral organoids to elucidate the mechanisms underlying the formation of CELs after irradiation [20]. Here, we show that cerebral organoids irradiated with C-ions in the SOBP exhibit similar responses to organoids exposed to X-rays and protons, including growth retardation, and formation of fluid-filled cavities [20].

We attribute dose-dependent growth retardation of young organoids to the sensitivity of progenitor cells. The irradiation of organoids with C-ions at d20, when they are comprised of neuroepithelial progenitor cells [18,31], was performed with lower doses (0.5–4 Gy) compared to the X-ray irradiation (1–8 Gy) due to the higher RBE of C-ions. Previous studies have demonstrated that human neural stem cells (NSCs) exhibit reduced proliferation and a dose-dependent increase in apoptosis in response to 0.5–10 Gy of C-ion irradiation [32–34]. The observed dose-dependent growth retardation in the exposed organoids likely reflects reduced cell proliferation and increased apoptosis, as demonstrated in our previous study [20]. This effect, together with radiation-induced formation of fluid-filled cavities, was observed starting from doses as low as 0.5 Gy of C-ion irradiation, further confirming the radiation sensitivity of naïve cell types.

Furthermore, the higher effectiveness and suitability of high-energy C-ions for tumor treatment compared to X-rays was demonstrated through lower doses of C-ions to achieve the same radiation response compared to X-rays. The RBE of 2 was observed for C-ions with respect to the endpoint of cavity formation in both organoids at day 60 (irradiated on day 20) and day 100 (irradiated on day 80), consistent with values previously determined for protons in the SOBP [20]. This strong correlation between radiation dose and the formation of morphological abnormalities indicates that the dose is more critical than radiation type (C-ions or protons) or the developmental stage of the organoids (day 60 or day 100) in determining the radiation response. It is noteworthy that cells within cerebral organoids are not fully differentiated by day 80, and that proliferating cells are retained beyond day 75 of culture [18], which may explain the consistent RBE of C-ions in fluid-filled cavity formation observed at both day 60 and day 100. Late effects of cranial radiotherapy in vivo are associated with the formation of fluid-filled edema due to endothelial cell damage [35]. We acknowledge that the organoid model lacks vasculature, immune cells, and the ability to model long-term in vivo responses, which limits direct extrapolation of the endothelial compartments commitment to whole-brain irradiation effects. However, despite the organoid model is deprived of the vasculature, the newly observed effect of aberrant choroid plexus formation in organoids is independent of the vascular system and mechanisms behind the development of its epithelium can be studied isolated from the vasculature in such a model. A morphological similarity between the exposed organoids and the choroid plexus organoids described by Pellegrini et al. [36] was observed. This was verified by the expression of cortical hem markers (*MSX1* and *LMX1*) preceding the formation of the CP [22], CP lineage markers (*OTX2*) [23], CP-related markers (*ZO1*, *AQP1*, *CLDN3*, *IGF2,* and *KIR7.1*) [24–26] at the mRNA level at d80 (60 days post-irradiation). ZO1, CLDN3, and AQP1 are integral components of the choroid plexus (CP) in vivo (reviewed in [24]). Additionally, IGF2 and KIR7.1, both expressed in the CP epithelium, play crucial roles in the regulation of CSF secretion [25,36]. An increase, but not statistically significant, in the expression of these markers predominantly after 1 and 4 Gy, and mostly no increase after 0.5 Gy compared to non-irradiated controls suggests that C-ions induce CP epithelium in dose-dependent manner.

The key markers of differentiation, including members of NOTCH (*NOTCH 1*, *NOTCH2, NGN2*, *HES1* and *HES5*), and WNT (*WNT3* and *WNT5a*, *LEF*) signaling are responsible for the maintenance of neural progenitor cells. Together with and BMP (*BMP4*) signaling, precisely regulated expression of these markers gives rise to the cortical hem that precedes the formation of the CP [22,23,30]. Although the expression levels of WNT3, WNT5a, and LEF1 can be upregulated in response to X-ray or proton irradiation, irrespective of the maturation status of organoids [20], C-ion irradiation led to a decrease, however not statistically significant, in the mRNA level of these markers. This downregulation may reflect differences in the pattern of DNA damage or signaling dynamics induced by densely ionizing C-ions compared to sparsely ionizing X-rays or less densely distributed protons. For example, C-ions induce more complex and clustered DNA damage, and stronger activation of TP53 than X-rays or protons, which can antagonize WNT and block proliferation [37,38].

However, the formation of the choroid plexus is tightly regulated by components of the WNT signaling pathway and does not depend on its constitutive activation; in fact, sustained activation of canonical WNT signaling has been shown to inhibit CP development [23].

The formation of CP after irradiation with C-ions occurred at the expense of neurons, observed as a decrease in the expression of MAP2, which is in accordance with our previously published data for X-rays and protons [20]. Although, C-ions are discussed as a promising option for the treatment of some pediatric brain tumors (reviewed in [39]), a higher plasticity of the pediatric brain [40] could make it more susceptible to the occurrence of radiation-induced lesions. The RBE of C-ions in the SOBP for the formation of cavities with CP-like structure being 2.0 underscores the need to adjust normal tissue dose limits for this radiation quality.

While the lack of statistical significance—attributable to the limited number of independent experiments—warrants cautious interpretation, our observations suggest that C-ion irradiation elicits gene expression changes similar to those induced by X-rays and protons across different stages of organoid maturation, as reported in our recent study [20]. Particularly, immunofluorescence staining against AQP1 and CLDN3, two markers co-expressed only in the CP, supported the epithelial nature of the aberrant CP-like formation in organoids at d100. These parallels highlight the potential for shared molecular pathways underlying radiation responses, regardless of radiation quality.

## Conclusions

These results support the hypothesis that radiological changes following radiotherapy for brain tumors are caused by aberrant CP deposition. The effect is observed after both X-rays and charged particles, but the RBE in the SOBP is about 2.0 for both protons and carbon ions. These data should be taken into account when considering treatment planning for brain tumors particularly in pediatric patients, while the molecular mechanism suggests possible countermeasures to the post-irradiation brain injury.

## Supplementary Material

Supplementary material

## Figures and Tables

**Table 1.** Quantitative analysis of the dose-response curve for the percentage of organoids with liquid-filling cavities.

Irradiation	alpha (Gy-1)	RBE

X-rays, d20	25 ± 3	-
X-rays, d80	1.9 ± 0.4	-
Protons, plateau, d80	2.0 ± 0.7	1.1 ± 0.4
Protons, SOBP, d80	3.6 ± 0.5	2.0 ± 0.5
12C, SOBP, d20	50 ± 6	2.0 ± 0.3
12C, SOBP, d80	3.8 ± 1.5	2.0 ± 0.9

Data for X-rays and protons are described in the Supplementary Material of ref. [20].

## Data Availability

Research data are included into Mendeley data repository (https://doi.org/10.17632/4sspc9rbr6.1k). Other raw data are stored in an institutional repository and will be shared upon request with the corresponding author.

## Appendix A. Supplementary data

Supplementary data to this article can be found online at https://doi.org/10.1016/j.radonc.2025.111224.

## Abbreviations:

CELs: contrast-enhanced lesions
CP: choroid plexus
RBE: relative biological effectiveness
RN: radiation necrosis
