# Supplementary material for: Relative biological effectiveness and neural stem cell fate in carbon ion–irradiated human brain organoids
Source: Radiother Oncol. Author manuscript; Available in PMC 2026 Jul 30. (PMC13421752; doi:10.1016/j.radonc.2025.111224)
Supplement: Supplementary material [file NIHMS2189860-supplement-Supplementary_material.docx]

**Supplementary material**

**Materials and Methods**

**Human embryonic stem cell culture**

All experiments were conducted using the feeder-free human embryonic stem cell line WA09-FI (H9), which was utilized in accordance with §4 and §6 of the German Stem Cell Act (registry numbers 3.04.02/0125 and 3.04.02/0125-E01). This cell line was initially developed by Dr. James Thomson’s team at the University of Wisconsin. H9 cells, received from the WiCell Research Institute in Wisconsin, USA, at passage 23, were used between passages 43 and 53 for the experimental work. Cells were maintained routinely on Laminin-521-coated culture dishes (BioLamina, #600962, at 10 µg/ml) and cultured in mTeSR1 medium (STEMCELL Technologies), supplemented with penicillin (50 U/ml) and streptomycin (5 µg/ml) (Merck, #A2212). Passaging occurred every 3–4 days using ReleSR (STEMCELL Technologies, #05872). For passage, the culture medium was first removed, followed by a wash with 2 ml of PBS, and then a treatment with 200 µl of ReleSR. After discarding the reagent, cells were incubated at 37 °C for 2 minutes, allowing selective detachment of pluripotent cells. The detachment process was halted by adding pre-warmed mTeSR1 medium, and cells were reseeded at a density of approximately 1.0 × 10⁵ cells/cm².

**Generation of cerebral organoids**

Cerebral organoids were generated following previously established protocols [1]. In brief, embryoid bodies (EBs) were initiated by detaching H9 cells with ReLeSR (Stemcell Technologies) for 3 minutes at 37 °C. A total of 18,000 cells were seeded per well into 96-well U-bottom suspension plates (Sarstedt), pre-treated with an anti-adherence solution (Stemcell Technologies). Cells were plated in embryoid body medium (EBM) supplemented with 4 ng/ml basic fibroblast growth factor (bFGF) and 50 µM ROCK inhibitor (Tocris Bioscience) and centrifuged for 5 minutes at 1,000 rpm at room temperature. On day 3, the medium was replaced with EBM lacking added growth factors. By day 5, EBs were transferred to 6-cm dishes (Sarstedt) containing neural induction (NI) medium to promote neuroepithelial tissue formation. Fresh NI medium was replenished every other day following transfer. On day 11, EBs were embedded in Matrigel (Corning) droplets and continued to be cultured in NI medium until day 13. At that point, the medium was switched to improved differentiation medium without vitamin A (IDM-A), supplemented with 3 µM CHIR99021 (Biovision), and refreshed every two days until day 18. From day 18 onward, EBs were transferred to T-25 flasks and maintained on an orbital shaker. On day 20, the culture medium was changed to improved differentiation medium containing vitamin A (IDM + A), with medium changes every 3–4 days. Finally, on day 40, 20 µl/ml Matrigel was added directly to the IDM + A medium.

**Irradiation of cerebral organoids**

At d20 or d80 of differentiation, 5–10 cerebral organoids in T25 suspension flasks were subjected to C-ion irradiation in a dose range of 0.5–4 Gy or 3–15 Gy. Samples were subjected to ion beams in a 40 mm Spread Out Bragg Peak (SOBP, LET: 50-105 keV/μm), representing the irradiation field of the tumor. Controls were sham-irradiated. The medium was exchanged immediately after irradiation or up to 1 h thereafter.

**Size measurements and quantification of cavity formation**

Organoid size was assessed by imaging 10 individual organoids at two different time points: day 20 (prior to irradiation) and day 60 (corresponding to 40 post-irradiation). Images were captured using a standard digital camera (Sony DSC-W220), and organoid areas were quantified using ImageJ software. Additionally, the number of organoids displaying visible cavities was recorded for organoids at day 60 (irradiated at day 20) and organoids at day 100 (irradiated at day 80), respectively, and expressed as a percentage of the total number analyzed.

**Immunofluorescence**

For immunofluorescence analysis, organoids were fixed in 3.7% paraformaldehyde (Carl Roth) at 4 °C overnight, followed by three 5-minute washes in PBS. Dehydration was performed using a graded sucrose series (7–60% sucrose in PBS; Sigma): organoids were incubated for 4 hours in 7%, 10%, and 40% sucrose, and overnight in 30% and 60% sucrose. Subsequently, organoids were embedded in a 7.5% gelatin (Neolab)/10% sucrose solution using custom 3D-printed PDMS molds. Embedded samples were snap-frozen on dry ice and stored at −80 °C before cryosectioning. Cryosections (10 µm) were obtained using a CM1860 cryostat (Leica Biosystems) and first treated with 0.5% Triton X-100 (ThermoFisher Scientific)/1% BSA (Carl Roth) in PBS for 30 minutes to permeabilize and block. An additional blocking was performed using 1% BSA in PBS for 30 minutes at room temperature (RT). Sections were then incubated with primary antibodies (AQP1 (Sigma Aldrich, #HPA019206, 1:500 dilution), Claudin 3 (Sigma Aldrich, #HPA014361, 1:400 dilution)) or MAP2-AF647 (Abcam, #ab225315, 1:300 dilution) diluted in 1% BSA/PBS for 1 hour at RT or overnight at 4 °C. After three PBS washes (5 min each), secondary antibody (anti-rabbit IgG (Thermo Fisher, #A-11012; 1:1000 dilution)) diluted in 1% BSA was applied for 1 hour at RT if unlabeled primary antibody was used. Nuclei were counterstained with 5 µg/ml DAPI for 4 minutes. Following DAPI staining, sections were washed twice with PBS and twice with Millipore water (5 min each), then mounted using fluorescence mounting medium (Dako). Imaging was performed on a Zeiss Axio Imager Z2 fluorescence microscope equipped with Metafer5 software (v4.3.12, Metasystems). Staining experiments were conducted on samples from three independent organoid preparations, with a minimum of three organoids analyzed per group. Image processing was carried out using ImageJ (v1.53i, NIH).

**Real-time RT-PCR analysis**

For each experimental condition, 3–5 organoids were collected and lysed in QIAzol Lysis Reagent (Qiagen, #79306). Total RNA was extracted using the RNeasy Mini Kit (Qiagen, #74106), following the manufacturer’s protocol, which included an on-column DNA digestion step using the RNase-free DNase Set (Qiagen, #79254). Depending on the downstream application, either 50 ng or 2 µg of total RNA was reverse transcribed using the RevertAid RT Kit (Life Technologies, #K1691).

Quantitative PCR was performed using the Hot FIREPol EvaGreen qPCR Mix Plus (Solis Biodyne, #08-24-0000S) on a QuantStudio 3 Real-Time PCR System (Applied Biosystems), with data analysis carried out using QuantStudio Design & Analysis software (v1.5.3). Expression levels were normalized to 18S rRNA, and human fetal and adult brain mRNA samples were included as reference controls. Primer sequences are provided in Supplementary Table 1.

**Statistical analysis**

Experiments were performed using organoid samples in triplicate (n = 3) for at least two independent experiments (N = 2). Where applicable (N = 3), statistical significance was determined using a threshold of p < 0.05. Statistical comparisons were performed using GraphPad Prism (v 9.3.1) for number of independent experiments (N) with a given number of organoids per experiment (n) as indicated in the figure legend and included Brown-Forsythe and Welch ANOVA with Dunnett´s post-test where applicable. Samples of organoids were randomly assigned to different treatments. No statistical methods were used to pre-determine samples sizes. Because of the nature of the treatment (irradiation), data collection and analysis were not performed blind to the conditions of the experiments.

**References**

1. Lancaster, M.A. and J.A. Knoblich, *Generation of cerebral organoids from human pluripotent stem cells.* Nature Protocols, 2014. **9**(10): p. 2329-2340.

**Supplementary Table 1. Primers for qRT-PCR**

| **Gene** | **Accession** | **Primer sequence (5´- 3´)** |
| --- | --- | --- |
| 18s rRNA | NR_003286.2 | ACTCAACACGGGAAACCTCACC (s) |
|  |  | CGCTCCACCAACTAAGAACGG (as) |
|  |  | TGTGTCGTGTTCTCAAAGGGT (as) |
| MSX1 | NM_002448.3 | CACTGAGACGCAGGTGAAGA (s) |
|  |  | CCAGCTCTGCCTCTTGTAGT (as) |
| LMX1A | NM_177398.4 | GGATGGTATTGTTCTGTGCTAGG (s) |
|  |  | GTGTTCAAGTCTCCAATGATGTCC (as) |
| OTX2 | NM_001270524.2 | CCCTCACTCGCCACATCTAC (s) |
|  |  | GGTTCAGAGTCCTTGGTGGG (as) |
| ZO1 | NM_003257.4 | CTCGCTCTCGGGAGATGTTT (s) |
|  |  | CTCCATTGCTGTGCTCTTGG (as) |
| AQP1 | NM_198098.4 | TGGACACCTCCTGGCTATTG (s) |
|  |  | GGGCCAGGATGAAGTCGTAG (as) |
| CLDN3 | NM_001306.4 | CCACGCGAGAAGAAGTACAC (s) |
|  |  | CCTGCGTCTGTCCCTTAGAC (as) |
| IGF2 | NM_000612.6 | CGCTGTTCGGTTTGCGA (s) |
|  |  | GATTCCCATTGGTGTCTGGAAG (as) |
| KIR7.1 | AB013891 | CCCACCTGAAAACCACACTACTG (s) |
|  |  | GCATGAGGCCTAGGAGCATTTG (as) |
| NOTCH1 | NM_017617.5 | CTGGTCAGGGAAATC GTG (s) |
|  |  | TGGGCAGTGGCAGATGTAG (as) |
| NOTCH2 | NM_024408.4 | TGTGACATAGCAGCCTCCAG (s) |
|  |  | CAGGGGGCACTGACAGTAAT (as) |
| NGN2 | NM_024019.4 | CGCATCAAGAAGACCCGTAG (s) |
|  |  | GTGAGTGCCCAGATGTAGTTGTG (as) |
| HES1 | NM_005524.4 | ATCCGGAGCTGGTGCTGATA (s) |
|  |  | TTGGTGATCAGTAGCGCTGT (as) |
| HES5 | NM_001010926.4 | TGAAGCACAGCAAAGCCTTC (s) |
|  |  | GAACTGCACGGCCTCCT (as) |
| WNT3 | NM_030753.5 | CCTCGCTGGCTACCCAATTT (s) |
|  |  | GCTGGGCATGATCTCGATGT (as) |
| WNT5a | NM_003392.7 | ATTCTTGGTGGTCGCTAGGT (s) |
|  |  | TCCTTGAGAAAGTCCTGCCA (as) |
| LEF1 | NM_016269.5 | CCCATCACGGGTGGATTCAG (s) |
|  |  | TGAGGCTTCACGTGCATTAGG (as) |
| BMP4 | NM_001202.6 | CGCTACTGCAGGGACCTATG (s) |
|  |  | TCCATGATTCTTGACAGCCAAT (as) |
